# Second generation effects of larval metal pollutant exposure on reproduction, longevity and insecticide tolerance in the major malaria vector *Anopheles arabiensis* (Diptera: Culicidae)

**DOI:** 10.1186/s13071-020-3886-9

**Published:** 2020-01-07

**Authors:** Alexander C. S. N. Jeanrenaud, Basil D. Brooke, Shüné V. Oliver

**Affiliations:** 1 Centre for Emerging Zoonotic and Parasitic Diseases, National Institute for Communicable Diseases of the National Health Laboratory Service, Johannesburg, South Africa; 2 Wits Research Institute for Malaria, MRC Collaborating Centre for Multi-disciplinary Research on Malaria, School of Pathology, Faculty of Health Sciences, University of the Witwatersrand, Johannesburg, South Africa

**Keywords:** Transgenerational effects, Insecticide resistance, *Anopheles arabiensis*, Longevity

## Abstract

**Background:**

Members of the *Anopheles gambiae* complex breed in clean, sunlit temporary bodies of water. Anthropogenic pollution is, however, altering the breeding sites of the vectors with numerous biological effects. Although the effects of larval metal pollution have previously been examined, this study aims to assess the transgenerational effects of larval metal pollution on the major malaria vector *An. arabiensis*.

**Methods:**

Two laboratory strains of *An. arabiensis*, SENN (insecticide-susceptible) and SENN-DDT (insecticide-resistant), were used in this study. After being bred in water polluted with either cadmium chloride, copper nitrate or lead nitrate, several life history characteristics that can have epidemiological implications (fertility, apoptotic damage to reproductive structures, adult longevity and insecticide tolerance) were examined in the adults and compared to those of adults bred in clean water.

**Results:**

All metal treatments reduced fecundity in SENN, but only lead treatment reduced fertility in SENN-DDT. Cadmium chloride exposure resulted in apoptosis and deformation of the testes in both strains. After breeding generation F0 in polluted water, F1 larvae bred in clean water showed an increase in longevity in SENN-DDT adult females. In contrast, after breeding the F0 generation in polluted water, longevity was reduced after cadmium and copper exposure in the F1 generation. Larval metal exposure resulted in an increase in insecticide tolerance in adults of the SENN strain, with SENN-DDT adults gaining the greatest fold increase in insecticide tolerance.

**Conclusions:**

This study demonstrates that a single exposure to metal pollution can have transgenerational effects that are not negated by subsequent breeding in clean water. 
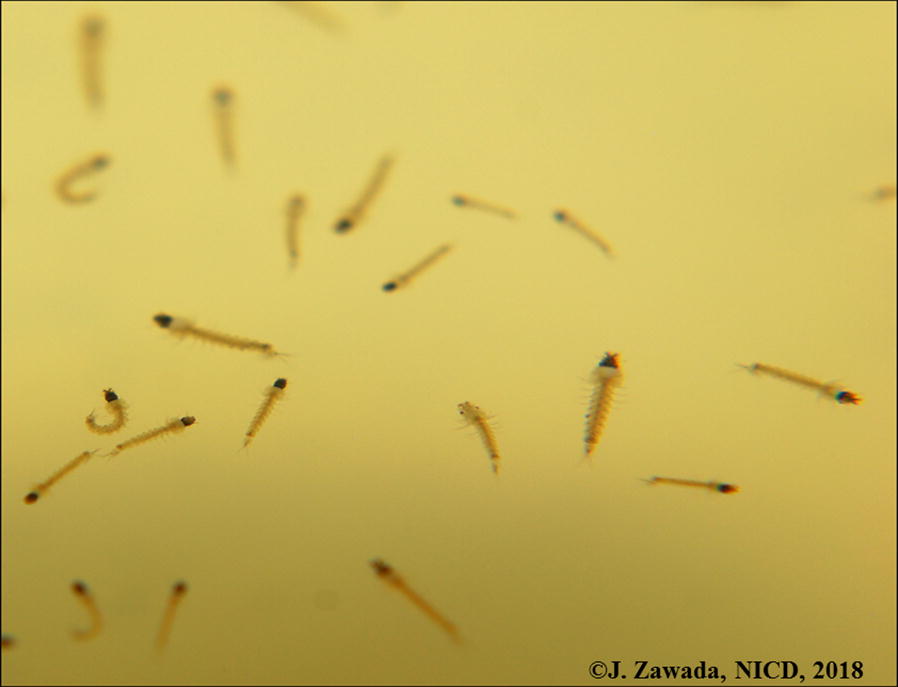

## Background

Heavy metals are persistent pollutants that are introduced into the environment by various anthropogenic activities [1]. These include mining, industrial and agricultural activities. Agricultural activities introduce heavy metals through the use of inorganic fertilizers [2], ultimately becoming contaminants associated with agricultural run-off [3–5]. This has implications for those invertebrate animals that breed in contaminated bodies of water [6–8].

The major malaria vector *Anopheles arabiensis* often breeds in bodies of water accruing from agricultural water run-off and has been shown to thrive on maize and rice pollen in particular [9, 10]. This is important in terms of ecological adaptation within this species because increased use of fertilizer and other agrochemicals has altered the chemical composition of these breeding sites [11].

A consequence of adapting to breeding in polluted water is that *Anopheles* malaria vectors are becoming better equipped to make use of sites that were previously inaccessible. This is resulting in range expansion, with reports of urban incidence of *An. arabiensis* in Cameroon and Nigeria among others, which was unreported until the 2000s [11–13]. The biochemical processes required to adapt to polluted environments have consequences for the mosquito populations and species concerned. Larval pollutant exposure has been demonstrated to increase adult insecticide tolerance in a range of mosquito species. It also affects larval development rate, and adult size and longevity, all of which have consequences for malaria transmission [14–17].

Previous studies on larval pollutants tended to focus on the exposure of a single generation, regardless of the pollutant [18, 19]. There have been studies that examined the transgenerational costs of selection to metal tolerance in *An. gambiae* [16, 17] but these studies are few. The transgenerational effects of larval metal pollutants on epidemiologically significant life history traits are poorly understood. Furthermore, adult female mosquitoes emerging from polluted waters may continue to use the same breeding sites or may choose alternative unpolluted waters. The transgenerational consequences of this breeding site choice are currently unknown. In this study, the transgenerational effects of larval metal pollution on two laboratory strains of *An. arabiensis* were assessed.

## Methods

### Strains

Two strains of *An. arabiensis* were used in this study. The SENN strain was colonised from Sennar, Sudan in 1980. From this strain, SENN-DDT was selected by continuous exposure to 4% DDT. The strain is still currently being maintained under selection pressure. It has been shown to display resistance to DDT, permethrin, deltamethrin, λ-cyhalothrin and malathion [20]. The resistance profile is due to a fixation for the L1014F mutation, as well as increased activity of glutathione S-transferase, cytochrome P450s and general esterases [21]. Mosquitoes were reared according to Hunt et al. [22].

For all experiments, the SENN and SENN-DDT laboratory strains of *An. arabiensis* were used. Larvae of these strains reared in metal polluted water constitute the F0 polluted generation. Fertility and fecundity experiments were performed on this group. The reproductive organs of this group were dissected and stained for apoptotic damage.

To examine the effects of pollution on the second generation, the emergent adults of this F1 polluted generation was split into two groups. The first group was the F1 polluted generation, where the offspring are reared in the same pollutant as their parental generation. The second group is the F1 clean (unpolluted) generation. The longevity of adults in these two groups was assessed and compared to the longevity of equivalent adults reared in unpolluted water. The insecticide tolerance of these two F1 groups was assessed, and compared to adults that emerged from untreated water, as well as to an F0 generation that emerged from polluted water.

### Effects of larval metal pollution on the fertility of the first generation

#### Effects of larval metal exposure on adult fertility and fecundity

Hatchlings (less than 24 h) of the SENN and SENN-DDT strains (*n* = 200) were reared in either clean water or water polluted with the maximum acceptable toxic concentration (MATC) of cadmium chloride (0.36 µg/l), copper nitrate (1.86 µg/l) or lead nitrate (4.39 µg/l) [23]. The MATC values were chosen as they are the highest concentration of these common pollutants legally allowed in the environment. This would therefore represent the lowest amount of selection pressure in a water body classed as polluted. These metal salts were chosen to continue the characterisation of the SENN and SENN-DDT strains begun in [24], and because they are among the most common heavy metals polluting water bodies in Africa [16, 25, 26].

**Fig. 1:**
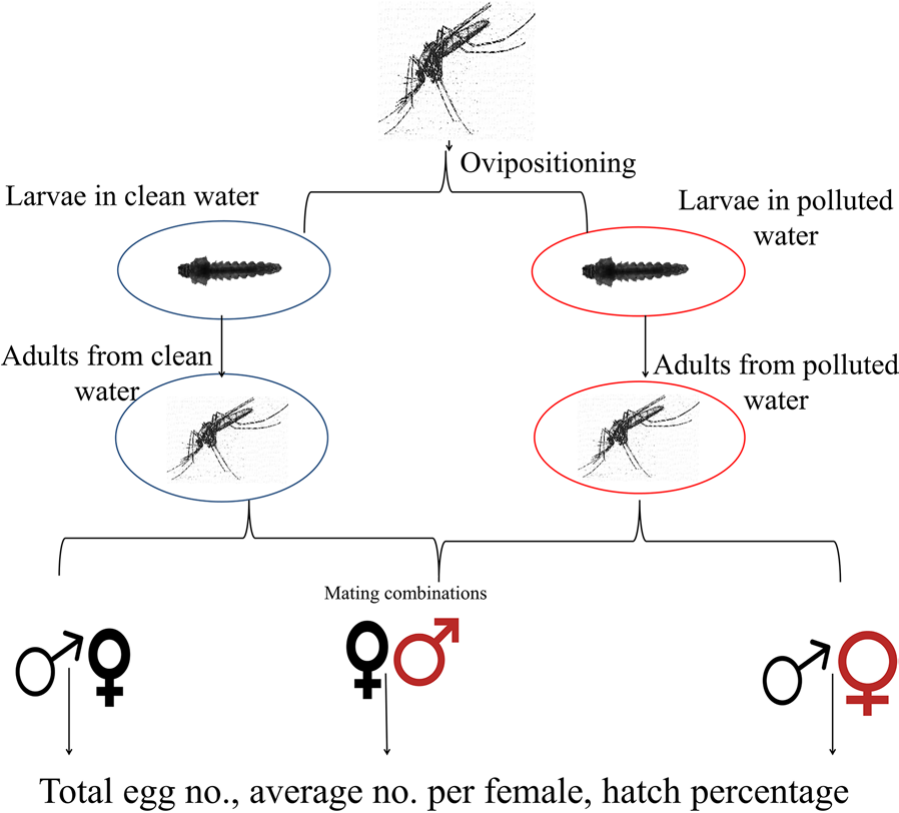
Schema of the mating experiments to determine whether male or female larval heavy metal treatment affects fertility or fecundity

Adults were allowed to emerge following which males and females were separated. Cross-mating experiments, each utilising 40 males and 20 females, were designed as follows: control male × control female, control male × metal-treated female, control female × metal-treated male (Fig. 1). A control with treated males mated with treated females was also performed. This treatment resulted in low egg numbers and no hatching. This is presented in Additional file 1: Table S1. Adult mosquitoes were allowed *ad libitum* access to 10% sucrose for the duration of the experiment. The females in all experiments were provided with a blood meal at the ages of 3 and 7 days, with an egg plate provided at day 11. The egg plate was removed 24 h after initial placement the eggs and hatched larvae were enumerated after a period of 4 days. This experiment was replicated 3 times using cohorts arising from separate egg batches.

#### Effect of larval metal exposure on adult reproductive organs

For all staining procedures, larvae were reared in heavy metals as described in the fertility and fecundity experiments. For testes observations, 1-day-old males (*n* = 25) from both SENN and SENN-DDT were anaesthetised and their abdomens removed. Females were allowed to mate and offered a blood meal at the age of 3 days. Their reproductive organs were dissected 72 h post-blood meal. Dissected reproductive organs were placed in sterile phosphate-buffered saline (pH 7.2) for further use. For egg isolation, 3-day-old adults (40♂ and 20♀) previously reared in heavy metals were blood-fed, egg-plated 48 h after the blood meal, and newly laid eggs were isolated for further use.

A solution of acridine orange, ethidium bromide and phosphate-buffered saline (PBS) was prepared from a 1 µl/ml working solution made up in turn from a stock solution of 5 mg/ml Acridine orange and 1 mg/ml ethidium bromide (a modified protocol of Abrams et al. [27]). Dissected samples were saturated on a glass slide with the solution and placed on a shaker at low speed for 7 min. Samples were subsequently viewed using fluorescent microscopy. An Olympus BX41 microscope equipped with a fluorescein isothiocyanate (FITC) at excitation wavelengths of 490–500 nm and a barrier filter at 510–530 nm with Stream Essentials^™^ software version 1.9.4 was used.

### Effect of first-generation larval pollution on adult life history of the subsequent generation

Females that emerge from polluted water can breed in the same polluted water or fly away and breed in clean water. The following experiments examined these two scenarios (a second generation breeding in polluted and clean water) on two crucial life history traits; longevity and insecticide resistance.

#### Effect of larval pollutant exposure on adult longevity of the second generation

SENN and SENN-DDT hatchlings (F0) were reared in metal water as described for the fertility and fecundity experiments. Control adults were reared in clean (unpolluted) water. Adults were collected and allowed to mate, provided with a blood meal at age 3 and 7 days, and allowed to oviposit at the age of 11 days. Eggs from these adults were reared in either clean water (F1 clean: F1C) or water treated at the MATC of the initial parental pollutant (F1 polluted: F1P). The adults that emerged were placed into separate cages with 30♂ and 30♀ each. Adults were given free access to sucrose solution and females were prevented from acquiring blood meals. Male and female mortality was recorded daily. This experiment was replicated three times from starting material originating from three separate egg batches. A Kaplan-Meier estimator [28] with log-rank tests [29] as a test for significance was used to analyse data.

#### Effect of larval pollutant exposure on adult insecticide tolerance of the second generation

Samples of larvae were reared in either cadmium chloride, copper chloride or lead nitrate treated water, from which adults representing an F0 generation were produced. Adults emerging from larvae reared in untreated water served as a control. This generation was allowed to breed and their eggs (F1 progeny) oviposited. Upon hatching, first-instar larvae were split into 2 groups: one was reared in clean water (F1C) and the other in water treated with the same heavy metal as that used to rear their F0 parents (F1P). Emerging F1 adults were collected and, at 3-days-old, were exposed to either malathion or deltamethrin by CDC bottle bioassay in order to determine lethal time to 50% mortality (LT_50_) [24]. In this way, SENN adults from treatment and control groups were exposed to dosages of 1 µg/ml of either insecticide for 0, 2, 4, 8, 16 or 32 min and SENN-DDT to 10 µg/ml for 0, 10, 20, 40 or 80 min. Unexposed adults served as environmental controls, and mosquitoes exposed to acetone served as a solvent control. Any experiment with control mortality exceeding 5% was discarded. LT_50_ was determined using Probit analysis [30].

### Statistical analyses

Dataset distributions were tested for normality using the Shapiro-Wilk test [31]. As the data for all replicates were normally distributed, differences in means were analysed using a 1-way analysis of variance with a 95% confidence interval, with Tukeyʼs HSD test used as a *post-hoc* test [32]. Variances in longevity curves were determined using the Kaplan-Meier estimator, with a log-rank test used as a measure of significance [28, 33]. All statistical analyses were performed using either IBM SPSS version 22 (IBM Corp. Released 2013. IBM SPSS Statistics for Windows, Version 22.0. Armonk, NY: IBM Corp) or Statistix 8 (Analytical Software, Tallahassee, Fl.).

## Results

### Effects of larval metal pollution on the fertility of the first generation

#### Effect of larval metal exposure on adult fertility and fecundity

**Fig. 2:**
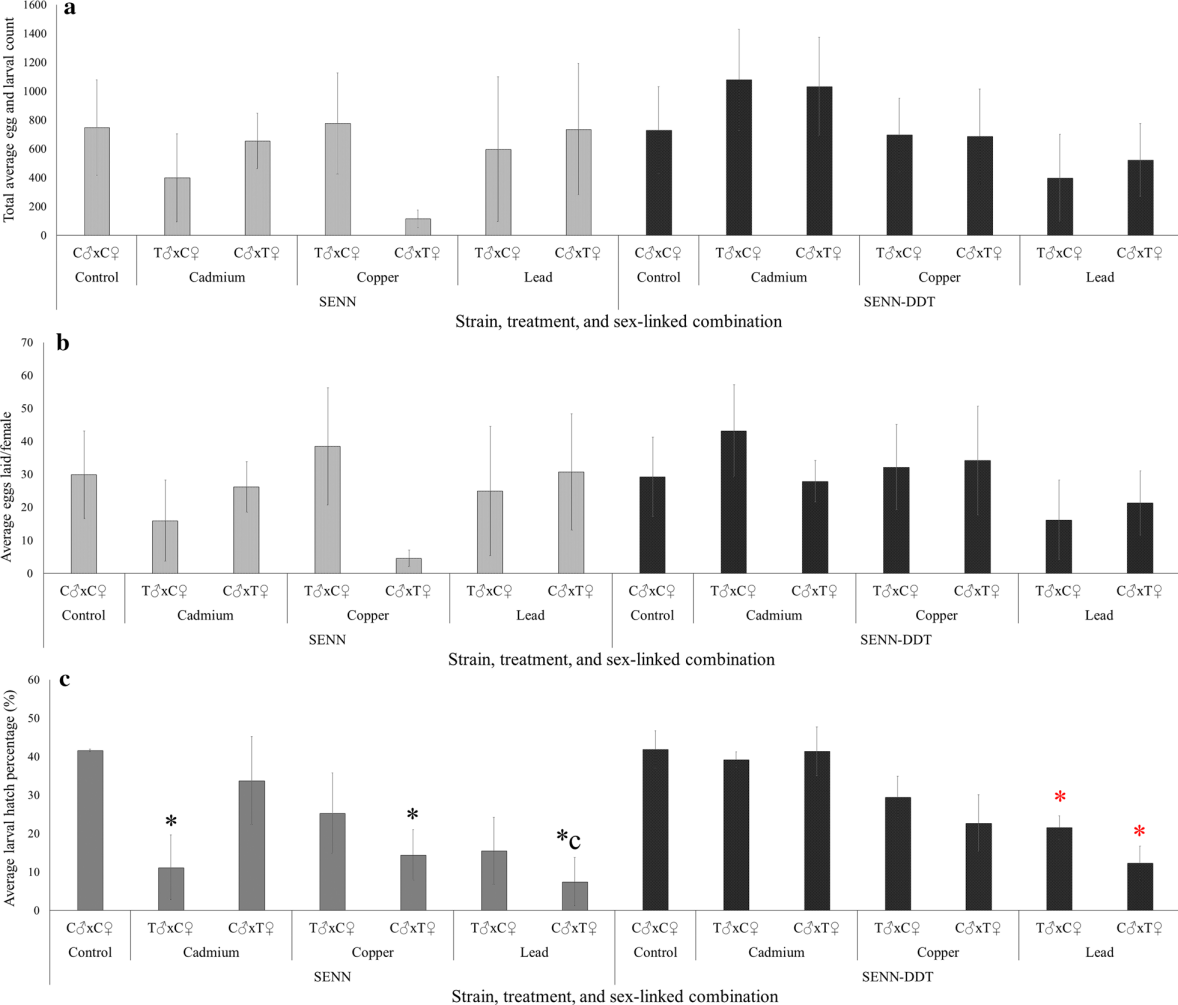
The effect of heavy metal exposure on the fertility and fecundity of *Anopheles arabiensis*. **a** The total number of eggs laid by SENN and SENN-DDT, where C represents control and T represents the respective metal treatment. **b** The average number of eggs laid per female by SENN and SENN-DDT, where C represents control and T represents the respective metal treatment. **c** Average hatch percentage off eggs laid by SENN and SENN-DDT. Asterisks indicate a significant difference from the control

The total number of eggs produced (fecundity) did not vary between metal treatments (Kruskal-Wallis One-Way ANOVA: SENN: *F*_(6, 25)_ = 1.01, *P* = 0.44; SENN-DDT: *F*_(6, 25)_ = 0.46, *P* = 0.82) (Fig. 2a). This was also true for the average number of eggs laid per female (One-Way ANOVA: SENN, *F*_(6, 25)_ = 0.54, *P* = 0.769; SENN-DDT, *F*_(6, 25)_ = 0.40, *P* = 0.871) (Fig. 2b).

In contrast, egg hatching (fertility) was significantly affected by larval metal treatment. For SENN, significant effects were evident in males treated with cadmium (2-sample t-test: SENN: control *vs* cadmium ♂: *t*_(8)_ = 3.62, *P* = 0.03) and females treated with lead (2-sample t-test: SENN: control *vs* copper: *t*_(8)_ = 4.13, *P* = 0.05; control *vs* lead ♀: *t*_(8)_ 5.45, *P* = 0.03). In SENN-DDT, significant effects were only evident following lead treatments in males and females (SENN-DDT: control *vs* lead ♂: *t*_(8)_ = 3.56, *P* = 0.02; control *vs* lead ♀: *t*_(8)_ = 4.46, *P* = 0.01). No other significant differences were evident in SENN (SENN: control *vs* cadmium ♀: *t*_(8)_ = 0.69, *P* = 0.53; control *vs* copper ♂: *t*_(8)_ = 1.57, *P* = 0.25; control *vs* lead ♂: *t*_(8)_ = 3, *P* = 0.09). This was also true for SENN-DDT (SENN-DDT: control *vs* cadmium ♂: *t*_(8)_ = 0.5, *P* = 0.64; control *vs* cadmium ♀: *t*_(8)_ = 0.05, *P* = 0.95; control *vs* copper ♂: *t*_(8)_ = 1.64, *P* = 0.16; control *vs* copper ♀: *t*_(8)_ = 2.17, *P* = 0.09) (Fig. 2c).

#### Effect of larval metal exposure on the cellular viability of adult reproductive structures

To understand the cellular basis of the changes in fecundity (egg production), levels of apoptosis were assessed in ovaries, testes and eggs. No significant changes were observed in ovaries, some qualitative differences were observed in eggs, and changes were observed in the male reproductive organs.

**Fig. 3:**
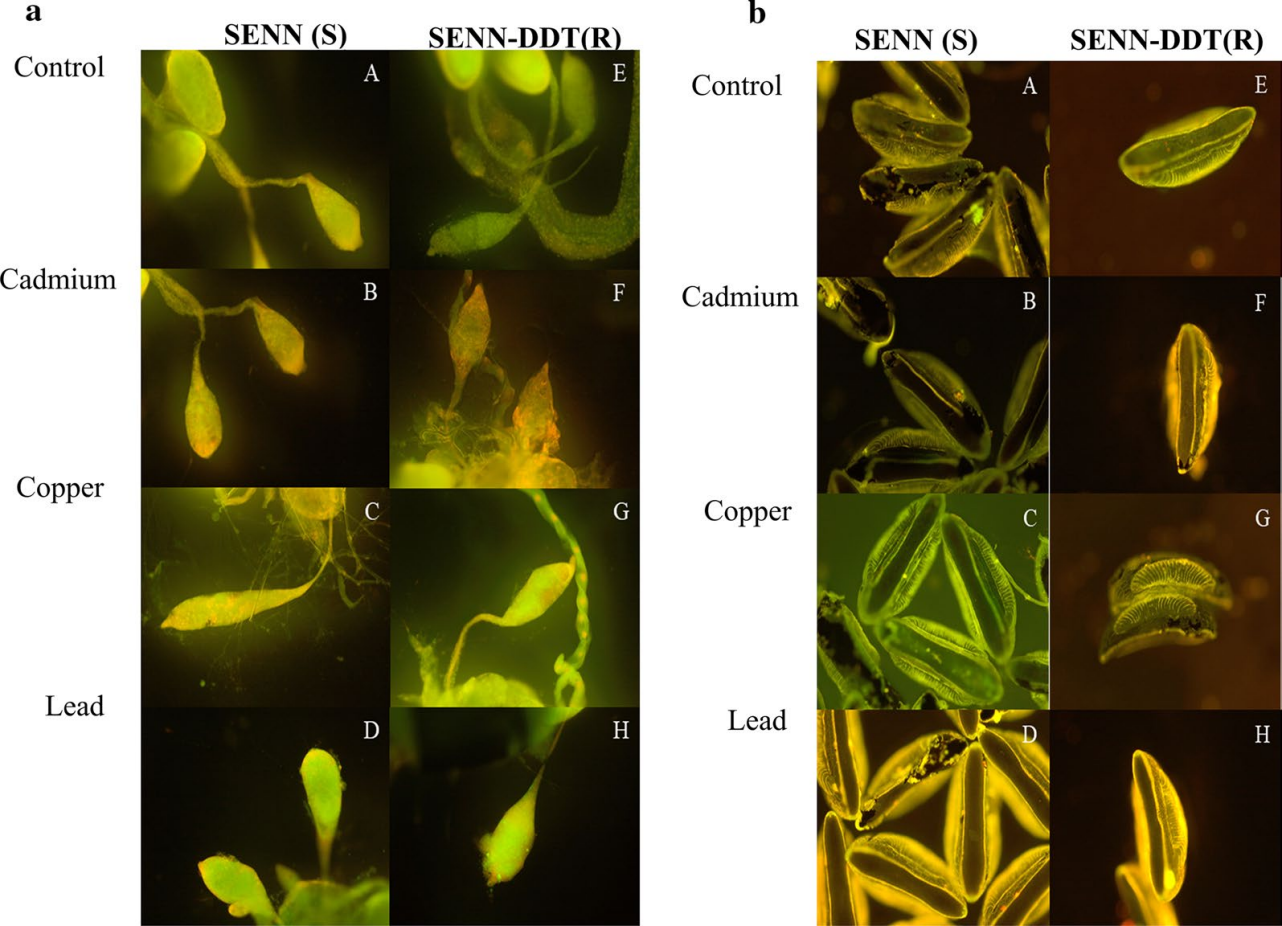
The effect of larval heavy metal exposure on apoptosis levels in the testes and eggs of *Anopheles arabiensis*. **a** The effect of larval exposure to metals on levels of apoptosis (indicated by red staining) in adult SENN (A-D) and SENN-DDT (G-H). **b** The effects of larval exposure to metals on oviposited eggs from SENN (A-D) and SENN-DDT (G-H)

In the untreated male samples, the testes and accessory glands showed green fluorescence without any clear signs of apoptosis in both strains (Fig. 3a, b). By contrast, larval metal exposure induced apoptosis in relation to toxicity i.e. cadmium, the most toxic metal, induced the greatest level of apoptosis (Fig. 3b, f), while lead, the least toxic, resulted in testes that resembled the controls (Fig. 3d, h). Copper generally induced greater levels of apoptosis in the insecticide susceptible SENN strain than in the SENN-DDT strain (Fig. 3c, g).

### Effect of first-generation larval pollution on adult life history of the subsequent generation

#### Effect of larval metal exposure on adult longevity of the second generation

**Fig. 4:**
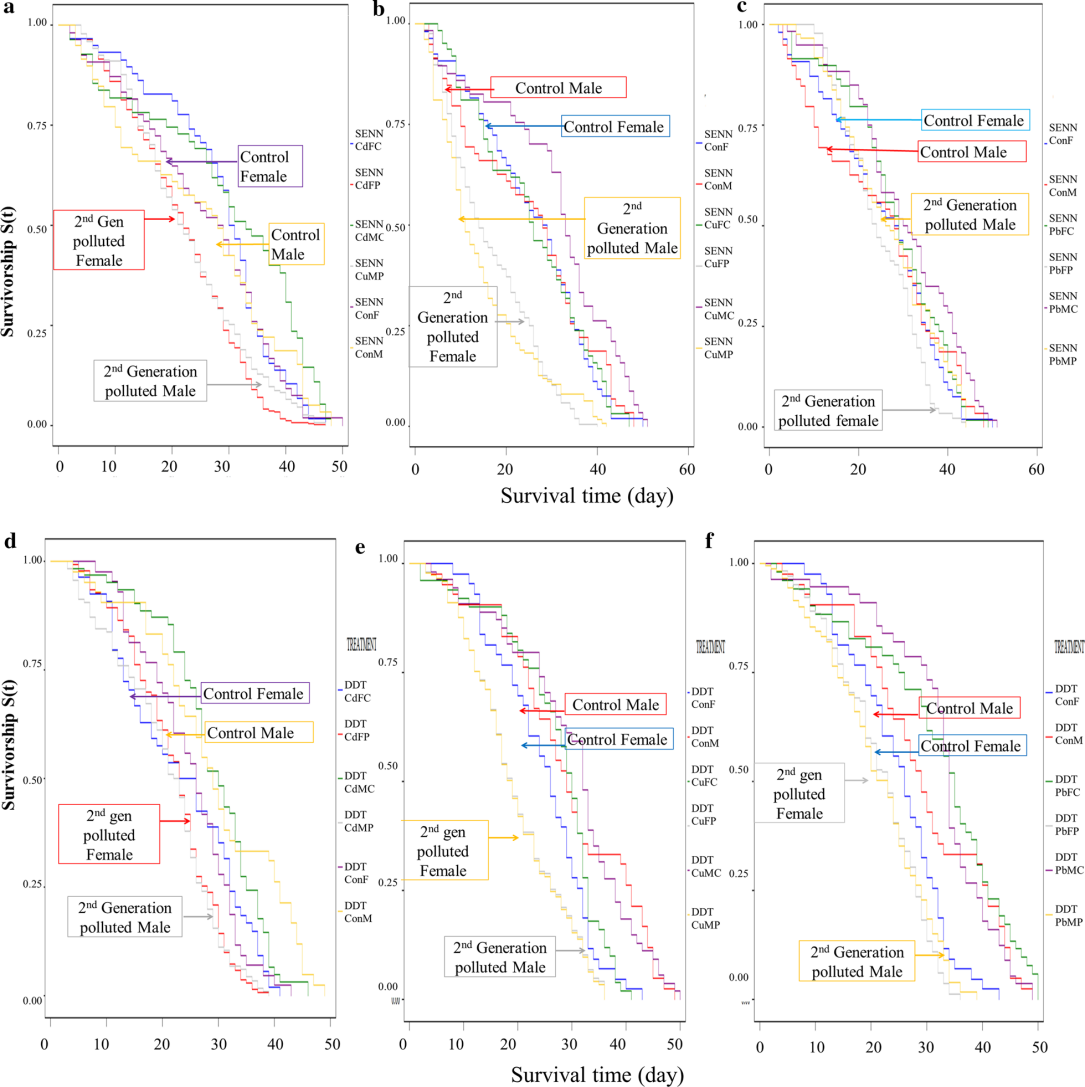
The effect of larval heavy metal exposure on parental (F0) and second-generation (F1) adult longevity in *Anopheles arabiensis*. **a** The effect of larval cadmium chloride exposure on a parental generation and second-generation (SENNConF- Control female, SENNConM-Control male, SENNCdFC-female cadmium second-generation from clean water, SENNCdMC-male cadmium second-generation from clean water, SENNCdFP- female cadmium second-generation from polluted water, SENNCdMP- male cadmium second-generation from polluted water) for the SENN strain. **b** The effect of copper nitrate exposure on a parental generation and second-generation for the SENN strain. **c** The effect of lead nitrate exposure on a parental generation and second-generation for the SENN strain. **d** The effect of cadmium chloride exposure on a parental generation and second-generation for the SENN-DDT strain. **e** The effect of copper nitrate exposure on a parental generation and second-generation for the SENN-DDT strain. **f** The effect of lead nitrate exposure on a parental generation and second-generation for the SENN-DDT strain

For the SENN strain, rearing in polluted water through two generations (F0 and F1) tended to reduce the longevity of F1 adults. This was true for cadmium (log rank test: *χ*^2^ = 74.94, *df* = 5, *P* = 0.00001), copper (*χ*^2^ = 121.94, *df* = 5, *P* = 0.00001),) and lead (*χ*^2^ = 19.94, *df* = 5, *P* = 0.01) treatments. This was also true for SENN-DDT cadmium (*χ*^2^ = 72.22, *df* = 5, *P* = 0.00001), copper (*χ*^2^ = 138.56, *df* = 5, *P* = 0.00001), and lead (*χ*^2^ = 138.90, *df* = 5, *P* = 0.00001), treatments. In general, the reduction of longevity in the SENN strain was greater than in the SENN-DDT strain (Fig. 4). Changes in LT_50_ are given in Table 1.

**Table 1:** Changes in *Anopheles arabiensis* adult median survival time [St(50)] (in days post-emergence) depending on breeding conditions/treatments

Treatment	SENN		SENN-DDT	
	Female	Male	Female	Male
Control	29	29	29	29
Cd-clean	23	34	31	36
change in St(50)	− 7	+ 5	+ 1	+ 5
Cd-Cd	15	12	23	22
change in St(50)	− 14*	− 17*	− 6*	− 7*
Cu-clean	26	34	30	33
change in St(50)	− 3	+ 5	+ 1	+ 5
Cu-Cu	15	11	19	19
change in St(50)	− 14*	− 18*	− 10*	− 10*
Pb-clean	30	31	35	31
change in St(50)	+ 1	+ 2	+ 6*	+ 2
Pb-Pb	24	27	24	23
change in St(50)	− 5	− 2	− 5*	− 6*

*Notes*: St(50), time to death of 50% of the population; metal-metal, F1 from polluted water reared in polluted water; metal-clean, F1 from polluted water reared in clean water

*Significant change from the control

*Abbreviations*: Cd, cadmium; Cu, copper; Pb, lead

#### Effect of larval metal exposure on the insecticide tolerance of a subsequent generation

Larval exposure to cadmium was associated with increased deltamethrin resistance in the F0 and F1 SENN generations, with the F1 generation showing further increases in insecticide resistance (One-way ANOVA: *F*_(3, 10)_ = 17.4, *P* = 0.00001). Copper exposure only showed increased deltamethrin resistance in the F1 generation (*F*_(3, 10)_ = 4.95, *P* = 0.02). Lead exposure induced increased resistance in the F0 and F1 generations, with resistance decreasing following F1 generation untreated water rearing (*F*_(3, 10)_ = 11.9, *P* < 0.0009).

**Fig. 5:**
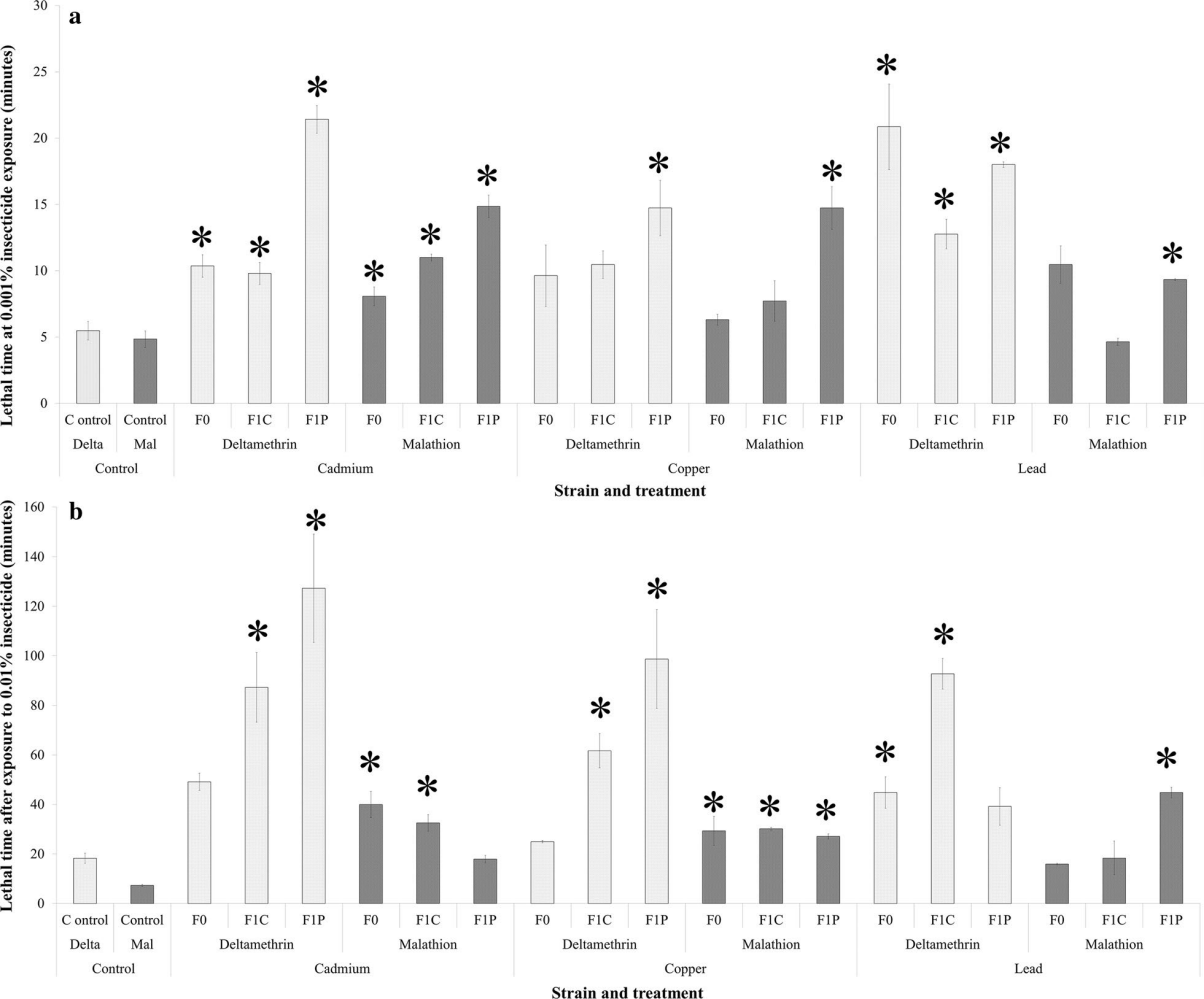
The transgenerational effects of larval metal pollutant exposure on insecticide tolerance in adult *Anopheles arabiensis*. **a** The effect of larval metal pollution on the deltamethrin (Delta) and malathion (Mal)-induced lethal times to 50% mortality (LT_50_) for the insecticide-susceptible SENN strain. The control, deltamethrin and malathion LT_50_s were determined from standard 3-day-old SENN non-blood-fed adults. This is compared to first generation metal exposed (F0) as well as second-generation adults reared from larvae bred in clean water (F1C) and second-generation adults reared from larvae exposed to the same pollutant as the parental generation (F1P). Asterisks indicate a significant difference in LT_50_ from the lethal time of the adults reared in clean water (control). **b** The effect of larval metal pollution on the deltamethrin (Delta) and malathion (Mal)-induced LT_50_s for the insecticide-resistant SENN-DDT strain. The control deltamethrin and malathion LT_50_s were determined from standard 3-day-old SENN non-blood-fed adults

SENN malathion resistance increased following cadmium exposures in both generations (*F*_(3, 10)_ = 39.8, *P* = 0.000001). Copper treatment only increased malathion resistance in the F1 generation (*F*_(3, 10)_ = 11.3, *P* = 0.0015). Lead exposure increased resistance to malathion in both generations (*F*_(3, 10)_ = 13.7, *P* = 0.0005) (Fig. 5a).

Larval exposure to cadmium was associated with increased deltamethrin resistance in the F0 and F1 SENN-DDT generations (unpolluted and pollutant) (*F*_(3, 10)_ = 13.6, *P* = 0.0005). This was mirrored by copper treatments (*F*_(3, 10)_ = 14.8, *P* = 0.0008). Lead treatments showed increases in resistance for both unpolluted (F0, F1C) generations, with a drop occurring in the F1 (polluted) generation (*F*_(3, 10)_ = 26.9, *P* = 0.00001).

Lastly, SENN-DDT cadmium increased malathion resistance after all three treatments (*F*_(3, 10)_ = 21.6, *P* = 0.0001) Copper treatments increased malathion resistance in all three exposures (*F*_(3, 10)_ = 14.6, *P* = 0.0008) and lead exposure only increased in the F1 generation (polluted) (*F*_(3, 10)_ = 25.7, *P* = 0.00001) (Fig. 5b). The fold changes are summarised in Table 2.

**Table 2:** Fold changes in *Anopheles arabiensis* adult lethal time to 50% mortality (LT_50_) by insecticide after different breeding conditions treatments/treatments

Generation and treatment	SENN		SENN-DDT	
	Deltamethrin	Malathion	Deltamethrin	Malathion
Gen 1 cadmium	1.89	1.67	2.69	0.73
Gen2C cadmium	1.79	2.27	4.77	0.46
Gen2P cadmium	3.91	3.06	6.96	0.21
Gen 1 copper	1.76	1.30	1.37	0.80
Gen2C copper	1.91	1.59	3.37	0.08
Gen2P copper	2.69	3.04	5.40	0.14
Gen 1 lead	3.80	2.16	2.45	0.04
Gen2C lead	2.33	0.96	5.07	0.93
Gen2P lead	3.28	1.93	2.14	0.29

*Abbreviations*: Gen 1, F0 bred in polluted water; Gen2C, F1 from polluted water bred in clean water; Gen 2P, F1 from polluted water bred in polluted water

## Discussion

As pollution, and in particular heavy metal pollution, becomes more prevalent it becomes increasingly important to understand its effect on the biology of vector mosquitoes. Metal pollution is a common genotoxicant affecting a number of organisms, including mosquitoes [34–36]. In terms of reproduction, it is unclear as to whether exposure to heavy metals affects males, females or both. From these data, heavy metal exposure significantly reduced fertility (number of hatchlings) but not fecundity (number of eggs produced). This was more marked in the SENN strain, where all metal treatments resulted in a reduced hatch. This was in opposition to SENN-DDT were only lead nitrate treatment resulted in a reduced hatch rate.

The basis of this reduction in fertility, however, remains unclear. The cross-mating studies indicated that in SENN, female exposure to copper nitrate and lead nitrate resulted in reduced fertility. In the SENN-DDT strain treatment of both males and females with lead nitrate resulted in reduced fertility. When examining apoptotic damage as a possible explanation for these findings, the ovaries were not compromised and there was little evidence of apoptotic damage in the eggs. Male testes were however compromised, with metals of greater toxicity inducing greater apoptotic damage (cadmium chloride > copper nitrate > lead nitrate). Qualitative analysis suggests that more apoptotic damage was sustained by the insecticide susceptible SENN strain. This corresponded to the patterns of reduced fertility. The mating experiments, however, only demonstrated a reduced fertility after male treatment of cadmium chloride in the SENN strain. Therefore, although it has been demonstrated that female treatments with heavy metals generally reduced fertility, no specific conclusions can be drawn. For males, it has previously been demonstrated, and is supported by the evidence presented here, that the testes are uniquely sensitive to cadmium-induced injury in many organisms (as reviewed in [37, 38]. This was clear in both strains where larval cadmium chloride exposure resulted in both apoptosis and deformation of the testes. Interestingly, although this resulted in a reduction in fertility in SENN, this was not mirrored in SENN-DDT where cadmium chloride exposure did not result in a significant reduction in fertility. This adds to the body of evidence that SENN-DDT is more metal-tolerant than SENN [24, 39].

Longevity is a crucial determinant of malaria transmission because a female mosquito needs to live long enough to become infective and thus epidemiologically significant. This is due to the 11–14 days extrinsic incubation period of the *Plasmodium* parasite [40]. Therefore, small changes in longevity can affect transmission [41]. For both strains, repeated breeding in polluted water significantly reduced adult longevity. This effect was more marked in SENN than SENN-DDT because the reduction in second-generation polluted longevity in the metal treatment groups was greater in SENN. In the SENN-DDT strain an initial lead nitrate exposure followed by breeding in clean water was associated with increased longevity, but this was negated by continued breeding in lead-polluted water. This suggests that continued breeding in polluted water could affect transmission intensity by reducing longevity, but that this effect appears more marked in insecticide-susceptible mosquitoes.

An important transgenerational effect of heavy metal exposure was the expression of insecticide tolerance. It has previously been demonstrated that larval exposure to metals in a single generation increases the expression of metabolic detoxification genes, cytochrome P450s in particular [42]. Similarly, single generation SENN and SENN-DDT larval exposures to metals increased the activities of detoxification enzymes in both strains [24]. There are also examples of the effect of metal induced selection for increased insecticide tolerance after ten generations [17]. Data from this study show that over and above a rapid, single generation increase in deltamethrin and malathion tolerance as previously demonstrated, this is carried through to the next generation. The rate of selection appears to be as rapid in the insecticide-susceptible SENN strain as in the SENN-DDT strain. Although the greatest fold increase in tolerance was seen in the SENN-DDT strain (cadmium-induced deltamethrin tolerance), the general pattern was that repeated rounds of pollution applied a greater selection pressure on SENN. In this strain every second round of pollutant exposure increased tolerance. This was not the case for SENN-DDT, highlighting the speed at which metal pollution can select for increased insecticide tolerance. It is worth noting that this rapid selection for insecticide tolerance coincides with a decrease in adult longevity. This highlights the detrimental effect of selection for insecticide tolerance on longevity as highlighted in previous studies [43]. Lastly, these data show that breeding in clean water does not reverse the effects of the initial pollutant exposures. In many cases, the second generation that was bred in clean water still had increased insecticide tolerance, or at least remained at the same level of tolerance as the initial exposure (except for SENN-DDT second-generation lead exposure after breeding in clean water and SENN second-generation after breeding in polluted water where tolerance was decreased). This is concerning as the effect of the pollutant exposure is therefore lingering and not negated by breeding in clean water.

There is a growing acknowledgement that increasing levels of larval pollutant exposure will affect vector biology and potentially alter transmission dynamics [44]. This includes range expansion into polluted environments and increased insecticide resistance levels [11, 24]. There is also evidence that pollutants alter the gut microbiome [45, 46] which could affect vector competence [47–49]. Added to these findings are the data presented here showing that single exposures to heavy metal pollution can have transgenerational effects on insecticide resistance, although these effects differed between the insecticide-susceptible SENN and insecticide-resistant SENN-DDT strains. SENN-DDT in general copes better in polluted environments, as previously demonstrated [24].

In general, adaptation to metal pollution is a complex, biologically costly process with variable effects on fitness [16]. Further studies, accompanied by field experiments, are required to understand the impact of pollution on adaptation and disease transmission, especially given that reductions in fertility and longevity may result in reduced rates of transmission. By contrast, increased insecticide tolerance, either accompanied or induced by increased tolerance to heavy metal exposure and associated with increased oxidative stress tolerance, may lead to increased malaria burden in changing environments [19, 20]. However, the question as to whether insecticide-resistant females are better or worse vectors than their susceptible counterparts remains, and contrasting arguments have been proposed [50–52].

## Conclusions

It is concluded that single exposures to heavy metals have transgenerational effects on *An. arabiensis*. This includes reduced fertility, possibly due to damage of male reproductive organs. Insecticide resistance amplifies after two rounds of larval exposure, but does not tend to drop below pre-exposure levels, regardless of the removal of the heavy metal selection pressure. Larval exposure to heavy metals can rapidly select for increased insecticide tolerance, but also tends to reduce adult longevity. This suggests that current levels of metal pollution may have biological consequences of epidemiological importance in *An. arabiensis* and that these effects are not immediately reversible.

## Supplementary information

**Additional file 1: Table S1.** Mean egg counts for male and females originating from polluted water.

## Data Availability

All data arising from this study is available within this article and its additional file.

## Abbreviations

ANOVA: analysis of variance
F1C: F1 clean
F1P: F1 polluted
MATC: maximum acceptable toxic concentration
PBS: phosphate-buffered saline
