# Supplementary material for: Second generation effects of larval metal pollutant exposure on reproduction, longevity and insecticide tolerance in the major malaria vector Anopheles arabiensis (Diptera: Culicidae)
Source: Parasit Vectors. 2020 Jan 7;13:4. doi: 10.1186/s13071-020-3886-9 (PMC6947826; doi:10.1186/s13071-020-3886-9)
Supplement: Supplementary file 1 — Additional file 1: Table S1. Mean egg counts for male and females originating from polluted water. [file 13071_2020_3886_MOESM1_ESM.docx]

**Additional file 1: Table S1: Mean egg counts for male and females originating from polluted water.** There was no significant difference between strains and treatment. None of the eggs hatched

|  | **SENN (±SE)** | **SENN-DDT (±SE)** |
| --- | --- | --- |
| **Cadmium chloride** | 35(0.02) | 40(0.01) |
| **Copper nitrate** | 27(0.01) | 23(0.03) |
| **Lead nitrate** | 42(0.02) | 39(0.01) |
